# Latex Metabolome of *Euphorbia* Species: Geographical and Inter-Species Variation and its Proposed Role in Plant Defense against Herbivores and Pathogens

**DOI:** 10.1007/s10886-021-01274-x

**Published:** 2021-04-21

**Authors:** Luis Francisco Salomé-Abarca, Dejan Gođevac, Min Sun Kim, Geum-Sook Hwang, Sang Cheol Park, Young Pyo Jang, Cees A. M. J. J. Van Den Hondel, Robert Verpoorte, Peter G. L. Klinkhamer, Young Hae Choi

**Affiliations:** 1 Natural Products Laboratory, Institute of Biology, Leiden University, Sylviusweg 72, 2333 BE Leiden, The Netherlands; 2 Institute of Chemistry, Technology and Metallurgy, National Institute, University of Belgrade, Studentski trg 12-16, Belgrade, 11000 Serbia; 3 Food Analysis Center, Korea Food Research Institute, Wanju, Republic of Korea; 4 Integrated Metabolomics Research Group, Western Seoul Center, Korea Basic Science Institute, Seoul, Republic of Korea; 5 College of Pharmacy, Kyung Hee University, 02447 Seoul, Republic of Korea; 6 Department Molecular Microbiology and Biotechnology, Institute of Biology, Leiden University, Sylviusweg 72, 2333 BE Leiden, The Netherlands; 7 Plant Ecology and Phytochemistry, Institute of Biology, Leiden University, Sylviusweg 72, 2333 BE Leiden, The Netherlands

**Keywords:** Metabolic constriction, Triterpenoids, Chemical defense, Mechanical defense, Chemical selection, 24-methylenecycloartanol

## Abstract

Based on the hypothesis that the variation of the metabolomes of latex is a response to selective pressure and should thus be affected differently from other organs, their variation could provide an insight into the defensive chemical selection of plants. Metabolic profiling was used to compare tissues of three *Euphorbia* species collected in diverse regions. The metabolic variation of latexes was much more limited than that of other organs. In all the species, the levels of polyisoprenes and terpenes were found to be much higher in latexes than in leaves and roots of the corresponding plants. Polyisoprenes were observed to physically delay the contact of pathogens with plant tissues and their growth. A secondary barrier composed of terpenes in latex and in particular, 24-methylenecycloartanol, exhibited antifungal activity. These results added to the well-known role of enzymes also present in latexes, show that these are part of a cooperative defense system comprising biochemical and physical elements.

**Supplementary Information:**

The online version contains supplementary material available at 10.1007/s10886-021-01274-x.

## Introduction

Living organisms are constantly challenged by diverse environmental factors, including biotic and abiotic stress (Andrew et al. 2010). In order to survive as a species, they must develop responses during these interactions, evolving genetically and/or phenotypically (Andrew et al. 2010). Therefore, the biological and chemical complexity of life can be considered to be, to some degree, the consequence of genetic diversity modulated by ecological interactions. Especially in plants, sessility has acted as a strong evolutionary pressure, resulting in the development of diverse physical and biochemical tools as sophisticated adaptive systems. This has allowed them to survive throughout time in diverse ecosystems (Bucharova et al. 2016). Among these tools, plant exudates have attracted great interest due to their adaptive origin, having resulted from their co-evolution with other organisms, including insect herbivores and microorganisms (Konno 2011). Plant exudates represent one of the surface defense layers associated with both primary and secondary strategies such as superficial physical structures (hairs, trichomes, thorns) and specialized metabolites, respectively (Kant et al. 2015). Among plant exudates, latexes have attracted much attention, not only because of their commercial value, but also due to their distinctive chemistry, which is assumed to possess ecological potential as a primary barrier in response to exogenous factors (Agrawal and Konno 2009).

Although the specific role of each individual latex metabolite remains unknown, as a whole latex metabolomes are clearly different from those of other organs of the plants exhibiting chemical fingerprints with both quantitative and qualitative differences (Konno et al. 2004; Konno et al. 2006; Seiber et al. 1982). While the role of individual latex metabolites remains largely unknown, as a whole there is undeniable evidence of their involvement in plant defense. For example, the concentration of bioactive metabolites of latex at damaged points before and upon an attack has been shown to be increased (Ball et al. 1997; Gorpenchenko et al. 2019; Hölscher et al. 2016). Latexes have also been recognized as reservoirs of defense-related enzymes (Konno 2011). Several cysteine and serine proteases, protease inhibitors, chitinases, lectins, and oxidases have been isolated and identified from these exudates (Pintus et al. 2010). These enzymes possess numerous physiological roles in signal transduction or oxidative defense systems (Gulsen et al. 2010; Usha-Rani and Jyothsna 2010). Therefore, gaining an insight into the role of latex metabolites and the factors that affect their chemical profiles could contribute to a greater understanding of chemical selection in the evolution of plant defense.

To explore the roles and mechanisms behind latex chemistry and its variations, a method was designed based on the following research questions. Do environmental factors influence the latex metabolome? If so, is this reflected in their role in plant defense system? And what is the outcome of this? Considering the multifactorial nature of the investigated phenomena, a holistic approach was considered to be the most appropriate. The experiments were performed on a set of samples of different genetic (species) and environmental (geographical origin) sources: wild *Euphorbia glareosa* Pall. Ex M. Bieb., *E. amygdaloides* L., and *E. palustris* L. (Euphorbiaceae) specimens collected in different locations in Serbia. The metabolic composition of latexes and tissues from the plants (leaves and roots) were studied by ^1^H NMR and LC-MS-based metabolomics, and GC-MS and HPTLC-DART-MS were used as supplementary tools for a more detailed analysis of targeted metabolites. Based on the results, the anti-herbivory, antibacterial, and antifungal activities of latexes, leaves and roots were tested. This defense might include both chemical and mechanical characters, the former due to a set of specialized metabolites, and the latter by provision of a physical barrier. The independent and complementary roles of these characters have never been explored and thus the effects of chemical extracts and a physical barrier, simulated by a layer of rubber, were tested separately. Subsequently, the degree of chemical and biological variation between samples was correlated in order to determine the roles of some individual metabolites. This holistic approach revealed the effect of environmental factors on the variation of the metabolome of latexes and its correlation with the plants’ defense systems.

## Methods and Materials

### Plant Material and Collection

Leaves, roots, and latexes of *Euphorbia glareosa* Pall. Ex M. Bieb., *E. amygdaloides* L., and *E. palustris* L. were collected in several locations in Serbia in June 2017. The samples of *E. glareosa* were collected at Deliblatska peščara (44°56′41.44”N 21°4′29.52″E), Zagajička brda 44°55′48.29”N 21°11′51.68″E), and Titelski breg (45°13′23.92”N 20°13′45.03″E). The samples of *E. amygdaloides* were collected at Avala (44°41′11.41”N 20°30′53.20″E), Mali Jastrebac (43°23′3.51”N 21°36′47.97″E), and Kosmaj (44°28′28.45”N 20°34′28.10″E). The samples of *E. palustris* were collected at Borča (44°54′48.34”N 20°26′32.51″E), Čenta (45°5′58.15”N 20°22′42.06″E), and Šajkaš (45°15′13.14”N 20°6′31.21″E). The plant material was identified by Pedja Janaćković, and voucher specimens were deposited at the Herbarium of the Botanical Garden “Jevremovac” University of Belgrade, Belgrade, Serbia (voucher numbers: *Euphorbia glareosa* (BEOU17303), *Euphorbia amygdaloides* (BEOU17306), and *Euphorbia palustris* (BEOU17304)).

Latex samples were obtained by making incisions in the plant stems with a sterile razor blade and collecting approximately 1 mL of latex in a sterile 2 mL-microtube containing 400 μL of MeOH. The samples were stored at −20 °C until they were freeze-dried. Leaf and root samples were manually collected from the plant and placed into plastic hermetic bags with silica gel and stored in an electric cooling box at −4 °C no longer than 8 hr. This material was subsequently stored at −20 °C until processed (not longer than 24 hr). The leaf and root samples were processed by grinding with liquid nitrogen and then freeze-drying. Dry leaf (10 g) and root (5 g) powders were sonicated with methanol during 15 min. After filtration, the resulting extract was partially dried using a rotary evaporator, and then taken to total dryness with a speed-vacuum drier.

### Test Organisms

Larvae of *Mamestra brassicae* were kindly provided by Pieter Rouweler from the Entomology Department at Wageningen University. *Pseudomonas putida* (NCCB26044) was purchased from The Netherlands Culture Collection of Bacteria. *Pseudomonas fluorescens* and *Pseudomonas viridiflava* were kindly provided by Dr. Paolina Garbeva (Kurm et al. 2019). *Alternaria alternata* (CBS 102.47) strain was purchased from the collection of the Westerdijk Fungal Biodiversity Institute, and *Botrytis cinerea* was kindly provided by Dr. Jan van Kan (Van Kan et al. 2017).

### Reference Compounds

β-Sitosterol, α-amyrin, β-amyrin, oleanolic- and usrosolic acid were purchased from Sigma-Aldrich (St-Louis, MO, USA). 24-Methylencycloartanol was previously isolated from the latex of *E. palustris* (Krstić et al. 2016).

### High Performance Thin Layer Chromatography-Direct Analysis on Real Time-Mass Spectrometry (HPTLC-DART-MS) Analysis

For thin-layer chromatography (TLC), latex extracts were diluted with methanol to a final concentration of 2 mg/mL. An automatic TLC sampler (ATS 4) (CAMAG, Muttenz, Switzerland) with a 25 μL Hamilton syringe was used to apply 30 μg of all of the samples as 6 mm bands on 20 × 10 cm HPTLC silica gel plates (60 F254) (Merck). The saturation time was 20 min, and humidity was set to 37% using a saturated MgCl_2_ solution. The samples were applied at 20 mm from the sides and 10 mm from the bottom of the plate. The distance between bands was 10 mm, resulting in 18 tracks per plate. Chromatographic development was performed in an automatic developer (ADC2) (CAMAG, Muttenz, Switzerland). The samples were separated with a mixture of toluene–ethyl acetate (8:2, *v*/v). The solvent migration distance was 75 mm from the application point. The HPTLC system was controlled by Vision Cats software.

For HPTLC-DART-MS analysis, each track of the plate was cut into 5 mm-wide strips using a smart glass plate cutter (CAMAG, Muttenz, Switzerland). The HPTLC strips were individually transported on a motorized rail to the ionization region. The plates were ionized with a DART ion source (Ion Sense, Tokyo, Japan) using helium gas (purity of 99.999%) at 450 °C and 3 L h^−1^. The plate scan speed was 0.2 mm s^−1^, and it was controlled with DART control software (Ion-Sense). The distance from the ion source to the plate was 1.5 cm. Detection was performed with an AccuTOF-TLC (JEOL, Tokyo, Japan) in positive ion mode. The TOF-MS was set with a peak voltage of 800 V and a detector voltage of 1900 V.

^1^H NMR analysis.

Freeze-dried latexes (5 mg) were re-suspended in 1 mL of CH_3_OH-*d*_*4*_ containing 0.418 mM hexamethyldisiloxane (HMDSO) as the internal standard, and ultrasonicated for 20 min. For leaf and root samples, 5 mg of the MeOH extracts were dissolved in 1 mL of CH_3_OH-*d*_*4*_. All solutions were centrifuged at 13,000 rpm for 10 min, and 300 μL of the supernatant was transferred into 3 mm-NMR tubes. The ^1^H NMR analysis was carried out with an AV-600 MHz NMR spectrometer (Bruker, Karlsruhe, Germany), operating at a proton NMR frequency of 600.13 MHz. For internal locking, CH_3_OH-*d*_*4*_ was used. All ^1^H NMR consisted of 64 scans requiring 10 min and 26 sec as acquisition time using the parameters of 0.16 Hz/point, pulse width (PW) = 30° (11.3 μs), and relaxation time of 1.5 sec. A pre-saturation sequence was used to suppress the residual water signal using low power selective irradiation at H_2_O frequency during recycle delay. The FIDs were Fourier transformed with exponential line broadening of 0.3 Hz. The resulting spectra were manually phased, and baseline corrected and calibrated to HMDSO at 0.06 ppm using TOPSPIN V. 3.0 (Bruker).

For quantitation of 24-methylenecycloaratanol, the ratio between its *endo* H-19 signal (δ 0.55) and the internal standard (HMDSO) signal (δ 0.06) was used in the formula: TISTr x HIST x [IST] / HTS, where TISTr = Ratio between target signal and internal standard signal, HIST = number of protons in the internal standard signal, [IST] = concentration of the internal standard in the sample, HTS = number of protons in the target signal. This formula provided concentration values expressed in μmol/5 mg of sample. This data was further transformed into μg/5 mg of sample and extrapolated to mg of 24-methylenecycloartanol per gram of latex.

### Gas Chromatography-Mass Spectrometry Analysis

Dried latexes (5 mg) were extracted with 1 mL of chloroform. The extract was taken to total dryness with a speed-vacuum dryer. The dried extracts were re-dissolved with 100 μL of pyridine by ultrasonication for 5 min. To this, 100 μL of BSTFA + TMCS (99:1, Supelco) was added, and the solutions were heated at 80 °C for 50 min. The solutions were then centrifuged at 13,000 rpm for 10 min, and the supernatants were transferred to micro-inserts for GCMS analysis on a 7890A gas chromatograph equipped with a 7693 automatic sampler coupled to a 5975C mass single-quadrupole detector (Agilent, Folsom, CA, U.S.A.). Separation was performed on a DB5 GC column (30 m × 0.25 mm, 0.25 μm thickness, JW Science, Folsom, CA, U.S.A.) with helium (99.9% purity) as the carrier gas at a flow rate of 1 mL/min. The initial oven temperature was 100 °C for 2 min, and then ramped at 10 °C/min to 270 °C, held for 1 min, ramped again to 290 °C at 5 °C/min for 15 min, and then to 300 °C at 5 °C/min and held for 3 min. The injector was set at 280 °C, and 1 μL of each sample was injected in splitless mode. The interface temperature was 280 °C, and the ion source and quadrupole temperature of the mass detector was 230 °C and 150 °C, respectively. Ionization energy in EI mode was 70 eV, and peaks were identified by comparison of the ion spectra with those in the NIST library (version 2008).

### Liquid Chromatography-Mass Spectrometry Analysis

Each latex sample (5 mg) was individually re-suspended in 1 mL of a methanol-water solution (80:20, *v*/v) and ultrasonicated for 15 min. The resulting extracts were diluted in a 1:10 (v/v) ratio to a final concentration of 0.5 mg mL^−1^. The samples were filtered with 0.20 μm regenerated cellulose membrane filters and analyzed using an Acquity UPLC HSS T3 column (2.1 mm × 100 mm, 1.7 μm; Waters, Milford, MA 01757). Samples were eluted with a gradient of 0.1% formic acid in water (A) and 0.1% formic acid in acetonitrile (B) starting at 10% B (0–30 min), 100% B (30–35 min), and 10% B (35–40 min) at 40 °C at a flow rate of 0.3 mL min^−1^.

MS detection was performed on a QTOF mass spectrometer (Bruker Impact HD) equipped with an electrospray ionization (ESI) source. The capillary voltage was 4000 V, and the drying temperature was 350 °C at 6 L min^−1^. The samples were analyzed in negative and positive mode in the range of 50–1200 Da. Data acquisition, alignment, peak picking, and neutral losses calculations were performed using Progenies QI software version 2.3 (Nonlinear Dynamics: a Waters Company, Newcastle, UK). Quality control (QC) samples consisted of a blend of all samples that was injected every five samples. Extraction solvent was injected as a blank. The data was normalized to total intensity and filtered by deleting mass features detected in blank samples at higher response levels than in latex samples. Data filtering resulted in 8015 mass features for data acquired in positive mode and 2064 features in negative mode.

### Metabolite Identification

Triterpenoid compounds were firstly identified by comparison of their mass spectra with those of the NIST library (version 2008) with a match higher than 85%. Moreover, their identification was supported by diagnostic ^1^H NMR signal assignments in the latex extracts and compared to those reported in literature. Finally, the presence of their molecular ion and corresponding adducts were used when detected in DART-MS to also support identification data. Molecular ion data from LC-MS data was compared to that of Dictionary of Natural Products for compound annotation. A threshold of 10 ppm was set as the mass error for possible matches.

### Preparation of Cis-1,4-Polyisoprene (Rubber) Solution

A sample of rubber (5 g; Sigma-Aldrich) was cut into small pieces and placed in 100 mL of chloroform. The rubber pieces were left in the solvent for 3 hr to swell, and then manually stirred every 20 min for 1 min until a semi-clear solution was obtained. The volume was then adjusted to 140 mL and stirred until a clear rubber slurry was formed.

### Anti-Herbivore Assays

The diet was prepared by mixing 28 g of agar, 160 g of cornflower, 50 g of beer-yeast, 2 g of sorbic acid, 1.6 g of methyl-4-hydroxybenzoate, 8 g of ascorbic acid, and 0.1 g of streptomycin per liter of water. The ingredients were added to warm water with continual stirring. The diet (15 mL) was placed in individual plastic containers and left to solidify at room temperature. For treatments, the dry methanol extracts were re-suspended in 2 mL of ultrapure water and ultrasonicated for 10 min (2X). These were added to the diets while semiliquid, manually mixed, and left to solidify at room temperature. The treatments consisted of methanol extracts of latexes, leaves, and roots. From the 10 samples from each location of each species, three random samples were mixed to form one composite sample. Therefore, three composite samples from each region of each plant species were obtained for the three tissues, resulting in nine replicates for each species of each tissue and three replicates for different locations. The final concentration of all of the treatments was 200 μg/mL. The negative control consisted of ultrapure water, and the positive control was a 200 μg/mL concentration of abamectin in the diet. The weight of the larvae was recorded after 5 d, and the weights of treated and untreated larvae (negative control) were compared.

### Antibacterial Assays

*Pseudomonas putida, P. fluorescens* and *P. viridiflava* were inoculated on Mueller-Hinton agar (MHA) plates and incubated overnight at 37 °C. From the overnight cultures, a single colony was used to inoculate 10 mL of Mueller-Hinton broth (MHB) and incubated at 37 °C with constant stirring (150 rpm). The bacterial suspensions were further adjusted with the addition of MHB to 0.5 of turbidity of the McFarland scale (10^6^ CFU/mL). The agar plates with treatments were prepared by filling 45 mm Petri dishes with 7 mL of MHA.

The broth microdilution method was used to determine the minimal inhibitory concentration (MIC) of the tested triterpenes according to the Clinical Laboratory Standards Institute guideline (NCCLS 2003). The compounds were dissolved in DMSO and diluted to reach final concentrations in the well from 512 μg/mL to 16 μg/mL, and then taken to a volume of 100 μl in each well with MHB. Each well was then inoculated with 50 μl of the 0.5 McFarland bacterial suspensions and incubated for 24 hr at 30 °C. The final concentration of DMSO in the well was 5%, which was also used as a negative control. A 100 μg/mL solution of spectinomycin in one well was used as a positive control. The bacterial growth was measured by optical density at 600 nm in a well microtiter plate reader (SPARK 10 M, TECAN). The MIC value was defined as the lowest concentration of a compound that completely inhibited the bacterial growth at 24 hr. All experiments were performed by triplicate. For *P. viridiflava*, the inoculations were done in nutrient agar 2 plates, and the assays were carried out in nutrient broth 2 at 28 °C.

To investigate the antibacterial effects of a layer of rubber, the agar plates were prepared by filling 45 mm Petri dishes with 7 mL of MHA (nutrient agar 2 for *P. viridiflava*). After medium solidification, 3 mL of rubber solution was poured over the medium and left to dry for 2 hr in a fume hood, resulting in a homogeneous rubber layer of ca. 0.05 mm of thickness. Finally, the plates were exposed for 10 min to UV light for sanitation. The negative controls were MHA plates without rubber covering. Four replicates were performed for treatments and controls of *Pseudomonas putida*, *Pseudomonas fluorescens*, and *Pseudomonas viridiflava*. The bacterial growth was evaluated at 24 hr and 48 hr. To determine whether bacteria were able to pass through the rubber layer to the agar, the rubber layer from the inoculated treatment plates was removed, and the plates were incubated for 24 hr at 28 ± 2 °C. Plates were then inspected for the appearance of bacterial colonies at the inoculation points.

### Antifungal Assays

To assess the antifungal effects of a layer of rubber, plates were prepared similarly to those used for antibacterial assays but using potato dextrose agar (PDA). Agar plugs (7 mm) with *Alternaria alternata* and *Botrytis cinerea* were placed on top of the rubber layer of the treated plates. The negative controls were PDA plates without a rubber layer, PDA plates just with rubber solution, and PDA plates with a combination of rubber solution and latex powder. For the latter, *E. palustris* and *E. myrsinites* were chosen as models due to their very different triterpenoid profiles. Five milligrams of all samples of each species were mixed to obtain a representative sample of each latex. The latex was then dissolved in the rubber solution to reach a concentration of 200 μg/mL. The plates were dried in the same way as in previous experiments. To observe if the fungi grew over the rubber layer or penetrated it, the layer was manually removed from the plate. Four replicates were performed for each fungal strain, and their growth and colony diameter were measured at 48 hr. The incubation temperature was 26 °C.

To test the antifungal activity of triterpenes contained in latex and some other common triterpernes, the Minimum Inhibitory approach was used. 24-Methylenecycloartenol and β*-*sitosterol, representative of different steps of the phytosterol pathway, and α*-*amyrin, β*-*amyrin, ursolic and oleanolic acids, as representative of different steps in the triterpene pathway, were selected to be tested against *Botrytis cinerea*. The compounds were dissolved in methanol and tested in two-fold dilution series from 2000 μg/mL until 62.5 μg/mL. The spore solution was adjusted to 2.5 × 10^5^ spores/mL in the well, and the final concentration of methanol in the well was 5%. The plates were sealed with a parafilm layer and incubated at 26 °C. The positive control consisted of nystatin in the same range of concentrations, and the negative control consisted of media with a final concentration of 5% of methanol in the well. The minimum inhibitory concentration (MIC) was defined as the minimum concentration in which there was total inhibition of fungal growth visible at 16 hr. The minimum effective concentration (MEC) was defined as the minimum concentration needed to see a degree of antifungal effect at 16 h. The plates were examined under a stereoscopic microscope at 16 hr intervals to observe any further effects on fungal growth.

### Combination Effect of Rubber and 24-Methylencycloartanol

To explore interactions between *cis*-1,4-polyisoprene (rubber) and triterpenes, 24-methylencycloartanol (20 mg), a representative latex triterpene, was added to 1 mL of slurry solution of rubber in chloroform (36 mg/mL). Separate tubes containing 20 mg of 24-methylencycloartanol dissolved in 1 mL of chloroform, and tubes containing only 1 mL of rubber solution were prepared as controls. All tubes were vortexed for 1 min and then taken to total dryness with a speed-vacuum dryer. Four replicates were performed for all conditions.

### Data Processing and Multivariate Data Analysis

The NMR spectra were bucketed using AMIX 3.9.12 (Bruker BioSpin GmbH, Rheinstetten, Germany). Bucket data were obtained by spectra integration at 0.04 ppm intervals. Peak intensity of individual peaks was scaled to total intensity and recorded from *δ* 0.20 to 10.02. Because of the residual signals of D_2_O and CH_3_OH-*d*_*4*_, regions of δ 4.75 – δ 4.9 and δ 3.28 – δ 3.34 were excluded from the analysis, respectively. Multivariate data analysis was performed using SIMCA P (v.15, Umeå, Sweden). Principal component analysis (PCA) and partial least square discriminant analysis (PLS-DA) were conducted for ^1^H NMR and LC-MS data. For PCA and PLS-DA analyses, the data was scaled using the unit variance (UV) method.

To assess the effect of the environmental factors and plant species on the metabolic variation of the samples, a soft independent model of class analogy (SIMCA) analysis was performed using geographical origin and plant species as PCA-classes separately in each plant species. In this model, local PCA models are separately constructed for each set class (geographic origin or plant species). The distances from each sample to each model are calculated and known as distance to the model (DModX). For species effects, the distances to the model (DModx) values were calculated setting each plant species as a PCA-class in the model, and geographical origin in each species to detect environmental effects in the separated models (only latexes, only leaves, and only roots). Data was scaled using the UV method, and the DModx values were transformed to their corresponding logarithm values. The logarithmic averaged DModx values (*N* = 30) ± standard error of each model was used as a measure of the strength of each factor in the chemical homogeneity of the samples. That is, the higher the DModX value, the higher the metabolic variation among samples.

In order to obtain and compare the total correlation of the effects of the geographical origin of the species on the chemical composition of the samples, PLS-DA modelling using UV scaling was also performed on each individual data set. The averaged *Q*^*2*^ from the permutation test (100 permutations) was used as a measure of the strength of the effects of the species and geographical origin on the chemical profile differences among the different plant tissues.

For the antifungal bioassays, the radial growth of the treatments was compared to their corresponding control by a Dunnett test setting the control sample as a control for the comparison of the treatments to the control and setting the rubber treatment as a control for its comparison to the latex supplemented treatments (*α =* 0.05) for *Alternaria alternata*, and after log transformation of the data for *Botrytis cinerea*. The anti-herbivory activity data variance, homogeneity, and mean comparison were done with a type 2 ANOVA test, and the mean comparison was performed with a least-square means test (*α =* 0.05) after log transformation using R software (V 1.1.456).

## Results

### Metabolic Variation in Euphorbia Latexes by Geographical Location

To compare the metabolic variation of each organ and latex, their ^1^H NMR spectral data were subjected to multivariate data analysis (MVDA). Firstly, the spectra of the samples from the three locations were analyzed by principal component analysis (PCA). As shown in Supplementary Fig. 1, the metabolome of all the profiled samples was distinctive for each *Euphorbia* species. This was predictable, as the species is a factor known to have a significant effect on the metabolome (Salomé-Abarca et al. 2018). Interestingly, however, the metabolic variation related to the geographical origin of latex samples showed much less variation than leaf and root samples. This is clearly visible in the PCA plot, that shows that latex samples are less or even not at all separated by their geographical origin while their corresponding leaves and roots samples are grouped accordingly (Supplementary Fig. 1).

**Fig. 1:**
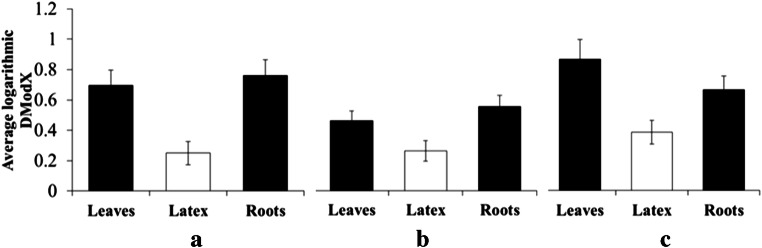
Logarithmic averaged distance to the model (DModX) values in latex, leaves, and roots of three Euphorbia species **a** *Euphorbia amygdaloides,* **b** *Euphorbia glareosa*. **c** *Euphorbia palustris*. The averaged values represent the mean (*N* = 30 ± standard error) of the DModX from a soft independent model of class analogy (SIMCA) analysis of all samples of leaves, roots, and latex per species. The DModX value is directly related to the metabolic variation among samples

However, considering that score distances on PCA can only be evaluated by visual inspection and are thus subjective, further MVDA was performed. A soft independent modelling of class analogy (SIMCA) analysis was constructed for the same sample set. For this model, a PCA is built for each set class, and the distance (DModX) between models is measured to determine their similarity or dissimilarity (Eriksson et al. 2011). In this model, the value of the DModX has a direct relation with the metabolic variation among samples. The values of DModX based on their geographical origin were calculated for latexes, leaves and roots of all species, and the obtained values were logarithmic-transformed and averaged to compare their degree of variation. As shown in Fig. 1, the SIMCA model confirmed that the variation in metabolomes among latexes from different geographical origins was much lower than that of leaves and roots for all of the tested species.

To further confirm this, the *Q*^*2*^ value (an indicator of correlation) of partial least square-discriminant analysis (PLS-DA) was calculated. The *Q*^*2*^ value reflects the degree of correlation between the chemical data set and the classes (Eriksson et al. 2011) (geographical origins) and can thus be regarded as a measurement of their degree of total correlation. Consequently, a lower degree of correlation could be anticipated between the latex chemical set and its geographical origins. As shown both by PCA and SIMCA results, geographical factors exert highly differential effects on the metabolic variation of leaves (*Q*^*2*^ = 0.96 ± 0.002) and roots (*Q*^*2*^ = 0.91 ± 0.012), while latexes showed much less correlation (*Q*^*2*^ = 0.53 ± 0.220), indicating that they are not affected as much by their geographical origin as their bearing tissues.

All the data analyses confirmed that the metabolome of latexes is more constant than those of leaves and roots. This apparent lack of influence of environmental factors results in a semi-conserved constitutive chemical pool that could act against a broad range of plant enemies. If this were the case, it could also be presumed that latexes are less influenced by the species factor than by geographical locations. Among the tested species, the metabolic variation in latexes (DModX = 0.76 ± 0.07) associated with the species was lower than in roots (DModX = 1.05 ± 0.08) but showed no relevant difference with that of leaves (DModX = 0.76 ± 0.06).

### Identification of Discriminating Metabolites

Comparison of the ^1^H NMR spectra of leaves, roots, and latexes revealed much higher levels of triterpenes in latexes than in the other tissues. The latex-specific triterpenes were characterized by methyl signals in the δ 0.70 – δ 1.90 range (Supplementary Fig. 2). Additionally, the cyclopropane moiety present in the structure of cycloartanol was identified by two doublets at δ 0.55 (d, *J* = 4.0 Hz) – δ 0.35 (d, *J* = 4.0 Hz) assigned to the 19-*endo* and 19-*exo* protons, respectively (Supplementary Fig. 3). The cycloartanols were further confirmed by DART-MS and GC-MS analysis. The direct mass analysis showed 441.4167 *m/z* and 458.4429 *m/z*, which were assigned to the [M + H]^+^ and [M + NH_4_]^+^ adducts (mass error < 7 ppm), respectively, of 24-methylenecycloartanol. In terms of the relative quantitation performed using integration of the H-19 resonance, it was found that the content of the cycloartanol-type triterpenes in latex was over eight times that of the plant tissues (Supplementary Fig. 3) accounting for almost 16% of the dry weight of *Euphorbia glareaosa* latexes, 30% of *Euphorbia amygdaloides* latexes, and 20% of *Euphorbia palustris* latexes. The GC-MS analysis confirmed this, revealing that a few other cycloartane analogues, such as 24-methylenecycloartanone and 24-methylenecycloartanol acetate, were more abundant in latexes than in other organs.

**Fig. 2:**
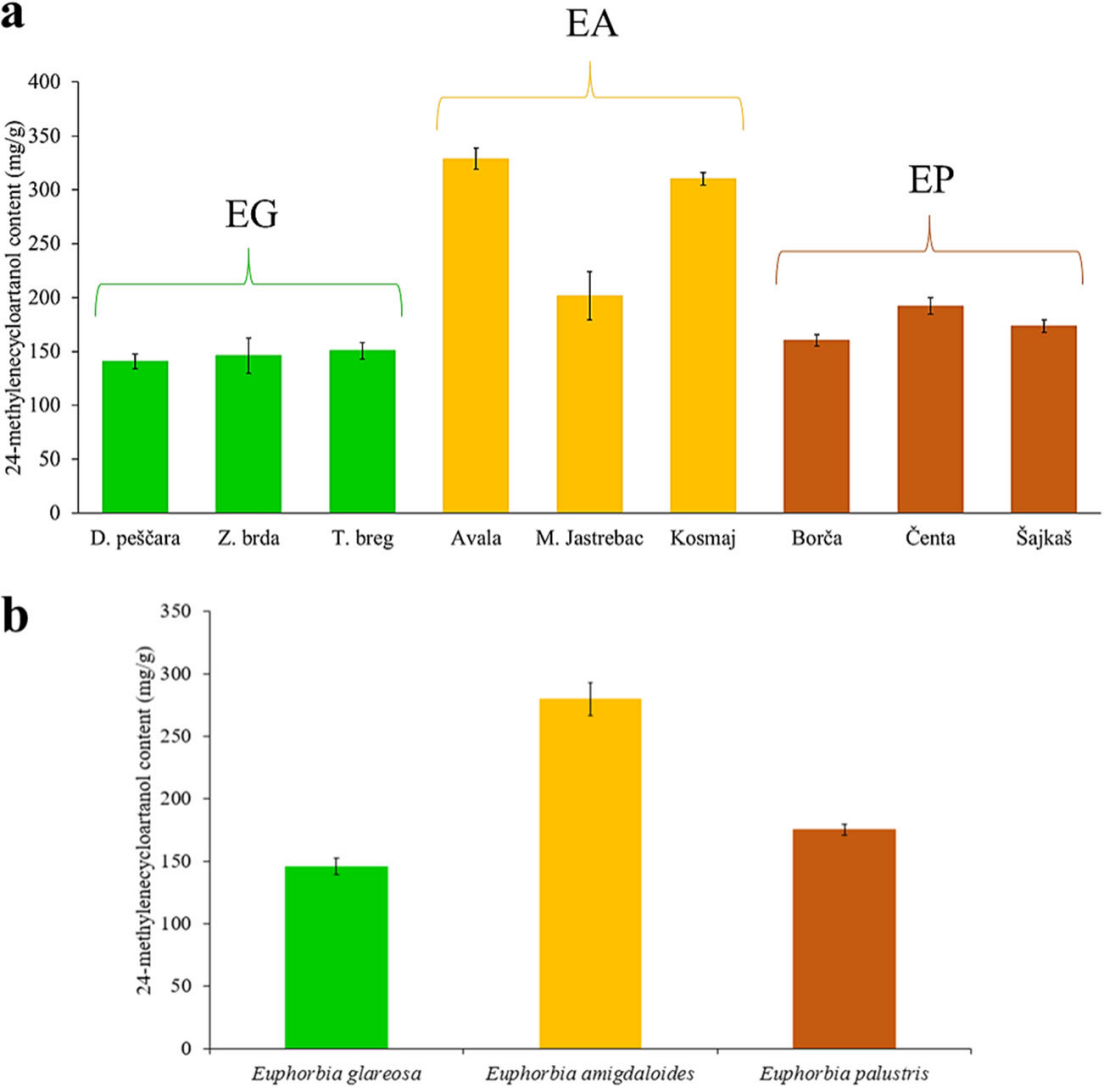
Variation of the content of 24-methylenecycloartanol in latexes according to: **a** geographical variation, and **b s**pecies (EG, *Euphorbia glareosa*, EA, *Euphorbia amygdaloides*, EP, *Euphorbia palustris*)

The triterpene 24-methylenecycloartanol was present in all analyzed latexes, but the variation of its content in samples from different geographical locations differed according to the species. For example, while *E. glareosa* and *E. palustris*latex showed no variation, latex from *E. amygdaloides* grown in one of the locations showed a lower concentration while the other two locations showed a similar content (Fig. 2a). However, averaging the samples from all locations, *E. amygdaloides* had the highest value while the other two species showed similar average content (Fig. 2b). The ^1^H NMR analysis also revealed the presence of another triterpenoid, cycloeucalenyl acetate, but this metabolite was specific for the latex of *E. palustris*. This compound was detected as two doublets resonating at δ 0.41 (*J* = 3.95 Hz, 1H) and δ 0.17 (*J* = 4.05 Hz, 1H) (Supplementary Fig. 4).

The LC-MS analysis of the latex samples provided further details of their metabolic variation. Similarly to the ^1^H NMR-based analysis, variation among latex samples from different locations was found to be low, and *E. palustris* and *E. glareosa* showed no grouping by geographical origin. However, several species-specific metabolites, such as alkaloids, were detected. Some alkaloids were found to be specific for *E. glareosa* and *E. amygdaloides*, while acyl sugars were determined to be more related to *E. palustris* (Table 1).

**Table 1:** Identified discriminant metabolites in *Euphorbia palustris, Euphorbia amygdaloides*, and *Euphorbia glareosa* latex collected in different locations of Serbia (± presence/absence of the compound; OM observed mass; ME mass error; IM ionization mode; Ns no cluster separation by PCA; Neg negative ionization mode; Pos positive ionization mode)

				*Euphorbia* Species								
Compound	OM	ME (ppm)	IM	*E. palustris*			*E. amygdaloides*			*E. glareosa*		
nicandrose E	650.3532	2.83	Neg	+			–			–		
sibiromycin^a^	648.3371	0.08	Neg	+			–			–		
asperazine	664.2791	−1.06	Neg	–			+			–		
milliamine C	706.2854	−5.14	Neg	–			+			–		
rankiniridine	552.2486	2.66	Neg	–			–			+		
				Geographical origin^c^								
				Bo	Če	Ša	Mj	Av	Ko	Tb	Dp	Zb
solanoglycosydane I	576.4099	−6.80	Neg	Ns	Ns	Ns	+	–	–	Ns	Ns	Ns
plactin	644.4008	−0.16	Neg	Ns	Ns	Ns	+	–	–	Ns	Ns	Ns
aphanamolide B	706.2830	−0.84	Neg	Ns	Ns	Ns	–	+	–	Ns	Ns	Ns
manadoperoxide J	406.7664	1.62	Neg	Ns	Ns	Ns	–	+	–	Ns	Ns	Ns
stelleralide C	666.2976	−9.55	Neg	Ns	Ns	Ns	–	–	+	Ns	Ns	Ns
C_36_H_36_N_2_O_8_^b^	624.2411	−0.08	Neg	Ns	Ns	Ns	–	–	+	Ns	Ns	Ns
pentaglycerol	388.1910	−8.76	Pos	Ns	Ns	Ns	+	–	–	Ns	Ns	Ns

^a^Derivative: 9-O-(4,6-Dideoxy-3-C-methyl-4-(methylamino)-α-L-mannopyranoside)

^b^Possibly grossamide, heliotropamide, or lyciumamide B

^c^Bo Borča; Če Čenta; Ša Šajkaš; Mj Mali Jastrebac; Av Avala; Ko Kosmaj; Tb Titelski breg; Dp Deliblatska peščara, Zb Zagajička brda

### Anti-Herbivore Assays

**Fig. 3:**
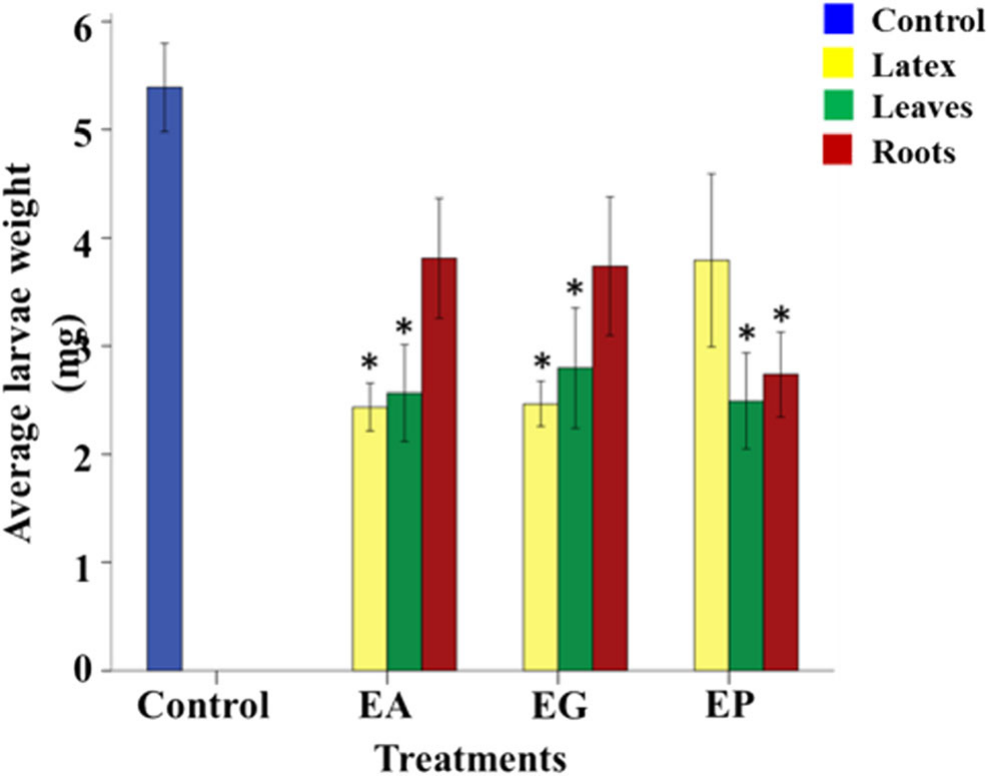
Anti-herbivore activity from latex, leaves, and roots of *Euphorbia amygdaloides*, *Euphorbia glareosa*, and *Euphorbia palustris*. The values represent the mean value (*N* = 30) ± standard error. The * represents significant differences between the treatment and the control in a least-square means test (α = 0.05). EA, *Euphorbia amygdaloides*, EG, *Euphorbia glareosa*, EP, *Euphorbia palustris*

Latexes, leaf, and root extracts of the three *Euphorbia* test species were challenged with a generalist herbivore, *Mamestra brassicae*. The results showed significant anti-feeding effects for all three types of extracts of two of the three tested species compared to the negative control diet (*P <* 0.05) (Fig. 3). In the case of *E. palustris*, the latex exhibited a lower feeding, but this was not statistically significant (*P >* 0.05), the large standard error being due to one outlier from Borča and one from Čenta, The variation of the anti-herbivore activity (standard error) of the latex was lower than that of leaves and roots in at least two of the three studied species (Fig. 3). This result suggested that the lower chemical variation of latexes was consistent with more homogeneous variation in biological activity against *M. brassicae*.

### Antibacterial Assays

When latex extracts were tested on *Pseudomonas viridiflava*, *P. fluorescens* and *P. putida*, considered to be general plant pathogens, they did not exhibit any antibacterial activity (*P* > 0.05).

In assays with a layer of rubber over the medium, none of the tested bacteria were able to move through the rubber layer (Supplementary Fig. 5) demonstrating, for the first time, that the mechanical defense of latexes could suffice to defend the plants from bacterial infections, or at least, that the barrier can delay their movement, and thus the onset of an infection.

### Antifungal Assays

**Fig. 4:**
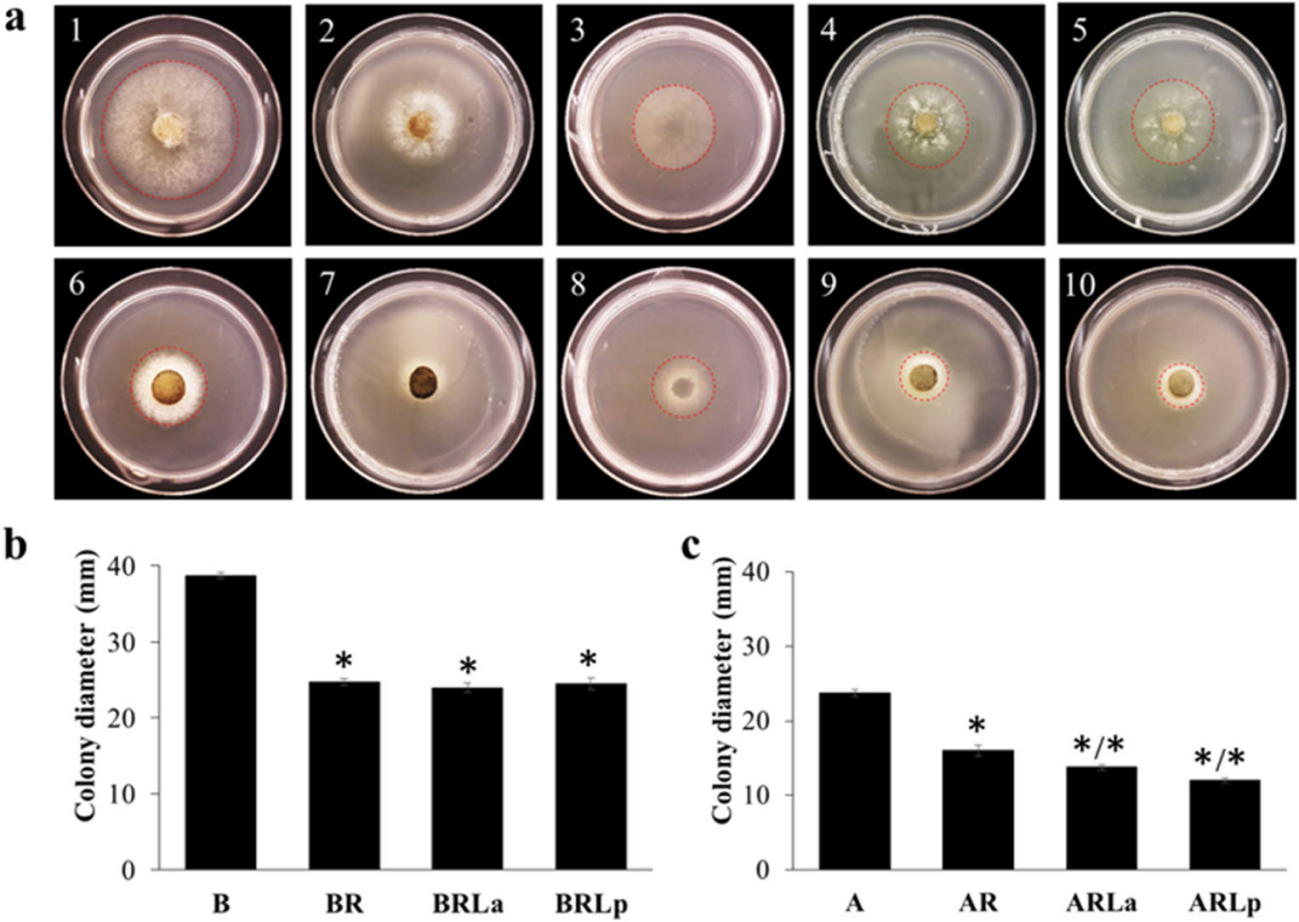
Antifungal activity of rubber and rubber plus latex against *Botrytis cinerea* and *Alternaria alternata* **a** Radial growth of *Botrytis cinerea* and *Alternaria alternata* in control and treated medium at 48 hr. **1**, Growth control of *B. cinerea* on PDA medium. **2**, *B. cinerea* growing on PDA medium covered with a rubber layer. **3**, Mycelium of *B. cinerea* in the agar medium after removing the rubber layer. **4**, *B. cinerea* growing on PDA medium covered with a rubber layer combined with 200 μg/mL of *E. palustris* latex. **5**, *B. cinerea* growing on PDA medium covered with a rubber layer combined with 200 μg/mL of *E. glareosa* latex. **6–10** represent the same treatment order, but against *Alternaria alternata.* **b** Antifungal activity of rubber and rubber supplemented with latex at 48 hr against *B. cinerea*. The values represent the mean (*N* = 4) ± standard error. The stars represent significant differences between the treatment and the control (*P* ≤ 0.05) in a Dunnett test; star after a slash indicates significant differences between the treatment the treatment with rubber only(*P* ≤ 0.05) in a Dunnett test. **B** growth control of *Botrytis cinerea*; **BR** covered only with a rubber layer; **BRLa**, covered with a rubber layer supplemented with latex of *E. amygdaloides;* **BRLp**, covered with a rubber layer supplemented with latex of *E. palustris*. **c** Antifungal activity of rubber and rubber supplemented with latex at 48 hr against *A. alternata*. The order of treatments is the same as for *B. cinera*,

A layer of rubber did not indefinitely block the growth of *Botrytis cinerea* or *Alternaria alternata* and the fungi were able to penetrate it (Fig. 4a). However, the radial growth of the colony submitted to the rubber treatment was significantly reduced relative to the uncovered control (*P* < 0.05) (Fig. 4a).

When the rubber slurry was supplemented with latex (Fig. 4b and c), for *A. alternata* there was a significant decrease (*P* < 0.05) in radial fungal growth in the latex- supplemented rubber compared to that observed with rubber alone, but not for *B. cinerea*.

The minimum inhibitory concentration (MIC) values of 24-methylenecycloartanol and other common steroids and terpenoids, including *β-*sitosterol, *α-*amyrin, *β-*amyrin, ursolic and oleanolic acids, were determined against *B. cinerea*. With the exception of latex-specific 24-methylenecycloartanol, none of the compounds showed inhibition at the tested concentrations. The MIC value of this triterpene was 2000 μg/mL, and the MEC value was in the range 500–1000 μg/mL. In contrast, some of the common steroids or triterpenoids displayed a growth-promoting effect on the fungus. For example, ursolic acid exhibited a strong growth-promotion effect in liquid suspensions of fungi 64 hr after inoculation. This indicated that 24-methylenecycloartanol plays a specific role in latex.

### Dispersive Effect of Polyisoprene

The MIC value of 24-methylenecycloartanol is too high to be considered a truly active fungicide. This low activity could be explained partially by its low solubility in the aqueous conditions of the test, and some component/s in the chemical matrix of the latex could act as a solubilizing agent. When a solution of pure 24-methylencycloartanol was dried, the compound crystalized in the bottom of the tube, whereas a mixture with polyisoprene formed a layer coating the wall of the tube and the sterol was determined to be evenly distributed throughout the layer (Supplementary Fig. 6).

## Discussion

The chemical composition of latexes includes three major categories of compounds: polyisoprene polymers (rubber), proteins, and low molecular weight specialized metabolites (Salomé-Abarca et al. 2019). Among these, proteins have been relatively more studied due to their evident biological functions against herbivores and pathogens (Konno 2011; Ramos et al. 2019). In the case of rubber, research has been focused on the coagulation process necessary for industrial purposes, but little is known about its role in defense mechanisms (Wahler et al. 2009). The chemical and biological diversity of latexes lies in the content of specialized metabolites. However, despite their proven biological activities, the potential individual or cooperative roles of these metabolites in the responses of the plants to the environment have not yet been fully explained. To gain a better insight into the nature of the defensive action provided by latex, their susceptibility to changes in environmental conditions was considered to provide essential information.

Due to their superficial anatomical location, latexes are very likely one of the first mechanical and chemical defenses of plants against herbivores and microorganisms (Castelblanque et al. 2016) and precisely because of this, should possess distinctive metabolomes. Furthermore, to be able to fulfil such a role regardless of the plant species, the latex metabolome should be composed of a few selected metabolites, resulting in a semi-conserved constitutive chemical composition that is relatively unaffected by environmental factors. To evaluate this possibility, a systematic approach examining latexes from plants from diverse locations was implemented with the aim of examining the chemical variation of latexes and roles of individual metabolites from plants from the same species but grown in different locations.

The latexes of three latex-bearing species of *Euphorbia*, collected in nine geographical locations, were metabolically characterized. The degree of metabolic variations of latexes, leaves, and roots of the plants was analyzed using several MVDA methods, including PCA, SIMCA, and PLS-DA. From the analyses, it was found that the metabolome of latexes clearly exhibited considerably lower metabolic variation per location than other parts of the corresponding plants. It is generally accepted that plant metabolomes are largely influenced by environmental factors; and the differences in chemical diversity in ecotypes have been well documented for some species and interpreted as an adaptive response to specific biotic and abiotic factors found in different ecosystems (Salomé-Abarca et al. 2020; Shelton 2004). Moreover, studies on leaf and root samples indicated that each plant species evolves differentiated chemical responses according to its survival requirements both at population and individual levels (Shelton 2004). Contrary to this general rule, the metabolome of latexes was shown to be relatively non- susceptible to changes caused by environmental factors. From a plant defense perspective, this lower metabolic variation of latexes indicates a chemical constitutive role in defense, deployed immediately before a more complex inducible response. Among the selectively accumulated chemical classes in *Euphorbia* latexes, triterpenes, and in particular, 24-methylenecyloartanol, was accumulated in latex rather than in the corresponding plant tissues. Moreover, its concentration was also shown to be quite unaffected by environmental factors and practically constant in all the analyzed *Euphorbia* species. The same applied for polyisoprenes. Several triterpenoids have been identified in *Euphorbia* species (Mahlberg and Pleszczynska 1983; Ponsinet and Ourisson 1968) and many of them have been suggested to be chemo-markers for certain taxons (Mahlberg et al. 1987; Mahlberg et al. 1988). Moreover, references show rubber and triterpenes as the main components of rubbery latexes independently of the latex plant-bearing species (Agrawal and Konno 2009; Konno 2011; Ramos et al. 2019; Salomé-Abarca et al. 2019). Also, it has been proposed that given their proximity in biosynthetic pathways, their coexistence in laticifers might have occurred during evolution (Mahlberg 1985; Nemethy et al. 1983; Piazza et al. 1987a, b). This hypothesis, however, does not explain the presence of specific terpenoids in concentrations approximately eight times that of other plant tissues, as observed in our results.

In considering latex as an integral defense system, the role of triterpenes in latexes should be functionally coupled to that of rubber. This would justify their lower degree of variation in latexes, strongly suggesting that their biological activities are mainly related, but not limited, to a constitutive and non-targeted broad protection in an early stage of defense.

To test this hypothesis, the anti-herbivory activities of latexes and the plant tissue extracts were assessed. Latexes, leaves, and roots exhibited almost the same degree of anti-herbivory effects against *M. brassicae*, despite possessing largely differentiated metabolomes. However, even with a more limited number of metabolites than those of their bearing plant tissue extracts, latexes exhibited a very effective metabolic composition designed against herbivores. That is, the anti-herbivory activity data of latexes against *M. brassicae* showed a lower variation (standard error) than leaves and roots samples, irrespective of their geographical location. Thus, these results suggested that the lower chemical variation of latexes was consistent with more homogeneous variation in biological activity against *M. brassicae*. Consequently, instead of a disadvantage, the low metabolic variation of latexes proved to provide a more stable and consistent protection against herbivores in diverse environmental niches. These results point to a long co-evolution between the plant species and diverse herbivores resulting in the selection of a determined or limited number of compounds to handle a broad range of natural enemies (Firn and Jones 2003). In particular, this conserved metabolome could be more beneficial for a constitutive defense role when dealing with generalist organisms, combining chemical and mechanical features. This is line with the generally accepted concept that when high herbivore pressure conditions are present, as in the case of latex-bearing species, the constitutive defenses could create a cost-benefit balance in plant fitness (Andrew et al. 2007; Moore et al. 2014). It is important to note that herbivores in these bioassays were exposed to equal dry masses of latex, leaf and root extracts. In natural conditions, it is more likely that herbivores will be exposed to smaller amounts of latex compared to those of leaves or root tissues. While this could imply that the experimental conditions were not entirely realistic, it is important to consider that the amount of exuded latex, depending on the coagulation speed, can vary from some drops to an almost freely flowing stream. Moreover, the quantity of exuded latex can rapidly increase, within minutes, just after herbivory and some insects could even be trapped in the flowing latex. Thus, all in all, the comparison made using the same dry weight comparison is biologically reasonable.

In nature, the most efficient way to deal with a wide range of natural enemies and to avoid the fast development of resistance with a minimal use of resources is to use a cooperative blend of traits (Richards et al. 2010; Richards et al. 2012). If this were the case for latexes, a possible complementary effect between their ingredients is to be expected. To examine this potential cooperative mechanism, the activity of polyisoprene (rubber) was firstly tested against bacteria and fungi. Rubber was found to block the invasion of bacteria completely. In the case of fungi, however, it worked partially, retarding the growth of the pathogenic fungi. This suggested the presence of metabolites that assisted the rubber barrier. Presumably, the hyphae of fungi that survived after penetrating the mechanical rubber barrier then came in direct contact with the latex inside the laticifer, which contains higher concentrations of active metabolites than the coagulated layer. This is where 24-methylenecycloartanol, by far the most abundant compound in the analyzed latexes, could act on the fungi. To test this idea, the fungicidal activity of this metabolite against *B. cinerea* was assayed and compared with other common terpenoids and steroids of leaves or roots. Among the tested compounds, only 24-methylenecycloartanol showed antifungal activity against *B. cinerea*. This indicated that 24-methylenecycloartanol plays a specific role in latex. Nevertheless, the isolated triterpenoid did not show strong effect on the growth inhibition of *B. cinerea* with relatively high MEC in the range of 500–1000 μg/mL. However, the low activity could be overcome by the natural presence in latexes as high as 11,000 μg/mL (Krstić et al. 2016).

These results reveal the existence of a systematic defense mechanism with a complementary effect between rubber and cycloartanol. This is supported by reports of the decrease in the expression of rubber synthesis genes when the expression of triterpene synthesis genes increases (Niephaus et al. 2019; Post et al. 2012). This was all reflected in the metabolome of the latex of *E. palustris* during fungal infection (Krstić et al. 2016). Thus, the literature and our results demonstrate that the selective accumulation and increase in concentration of 24-methylenecycloartanol in *Euphorbia* latexes is not random. That is, in the point of plant injury-fungal contact, rubber will retard the proliferation of the fungus and the hyphae that penetrate the latex barrier will encounter a high concentration of a specific antifungal component. This highlights how latexes initially deal with a large number of enemies with a very limited number of constitutive compounds.

Another aspect of the complementary mechanism involving cycloartanol and rubber is that rubber could function as a natural dispersive agent for the triterpenoids in latex. The results from the tests performed to demonstrate this, showed that the triterpene-rubber dispersion, rubber films, and their combination with latexes acted as a well-designed cooperative system against bacterial and fungal microorganisms. In this system, the rubber particles function as a carrier and disperser of phytosterols and triterpenes during latex exudation and throughout the whole production of a sealing film after coagulation. Therefore, the complementarity between the mechanical defense (rubber coagulation) and chemical defense (specialized metabolites) resulted in a polymeric film in which embedded metabolites are evenly distributed throughout the whole film to increase its effectiveness over the protected area. In addition to the fungicidal activity and function as a dispersion agent, triterpenes might have other functions. They could contribute to the polymeric structure of polyisoprene to strengthen the defensive barrier, as may be deduced from other related experiments (Mironenko et al. 2016; van Deenen et al. 2019). Further work is needed to understand the specificity of the synergy between latex and cycloartanol in particular, among many other available steroids and triterpenoids.

These findings show the complementation of mechanical and chemical features in a single defense system against herbivores, bacteria, and fungi. In normal conditions the metabolome of latexes presents a semi-conserved constitutive composition, consisting mainly in triterpenes throughout all *Euphorbia* species, with a very low susceptibility to environmental factors as shown by their low metabolic variation. This lower variation of selected active metabolites also guarantees a more constant and stable protection against herbivores. Rubber, one of the ubiquitous metabolites in latexes, showed that its contribution to the defense against bacteria, fungi, and herbivores (Dussourd 1995) is not limited to a physical trapping and blocking capability, but also acts a sort of drug delivery system, especially for terpenoids and probably other lipophilic latex metabolites. The defense system of *Euphorbia* latexes not only depends on metabolites released during latex exudation, but on their high concentration and potential overexpression in an inducible defense response after latex coagulation. The structural complementation between rubber and some specific metabolites, and the role of inducible metabolites related to rubber synthesis in the latex-borne defense is a promising horizon to explore for new complementary defense mechanisms of latexes.

## Supplementary Information

ESM 1 (DOCX 1860 kb)
