# Supplementary material for: Latex Metabolome of Euphorbia Species: Geographical and Inter-Species Variation and its Proposed Role in Plant Defense against Herbivores and Pathogens
Source: J Chem Ecol. 2021 Apr 21;47(6):564–76. doi: 10.1007/s10886-021-01274-x (PMC8217033; doi:10.1007/s10886-021-01274-x)
Supplement: Supplementary file 1 — (DOCX 1860 kb) [file 10886_2021_1274_MOESM1_ESM.docx]

# Supplementary Information

# Latex Metabolome of *Euphorbia* Species: Geographical and Inter-Species Variation and its Proposed Role in Plant Defense against Herbivores and Pathogens

**Luis Francisco Salomé-Abarca^1^, Dejan Gođevac^2^, Min Sun Kim^3^, Geum-Sook Hwang^4^, Sang Cheol Park^5^, Young Pyo Jang^5^, Cees, A. M. J. J. Van Den Hondel^6^, Robert Verpoorte^1^, Peter G. L. Klinkhamer^7^, Young Hae Choi^1,5, *^**

^1^ Natural Products Laboratory, Institute of Biology, Leiden University, Sylviusweg 72, 2333 BE Leiden, The Netherlands

^2^ Institute of Chemistry, Technology and Metallurgy, National Institute, University of Belgrade, Studentski trg 12-16, 11000 Belgrade, Serbia

^3^ Food Analysis Center, Korea Food Research Institute, Wanju, Republic of Korea

^4^ Integrated Metabolomics Research Group, Western Seoul Center, Korea Basic Science Institute, Seoul, Republic of Korea

^5^ College of Pharmacy, Kyung Hee University, 02447 Seoul, Republic of Korea

^6^ Department Molecular Microbiology and Biotechnology, Institute of Biology, Leiden University, Sylviusweg 72, 2333 BE Leiden, The Netherlands

^7^ Plant Ecology and Phytochemistry, Institute of Biology, Leiden University, Sylviusweg 72, 2333 BE Leiden, The Netherlands

^*^ Corresponding author

E-mail: [y.choi@chem.leidenuniv.nl](mailto:y.choi@chem.leidenuniv.nl)


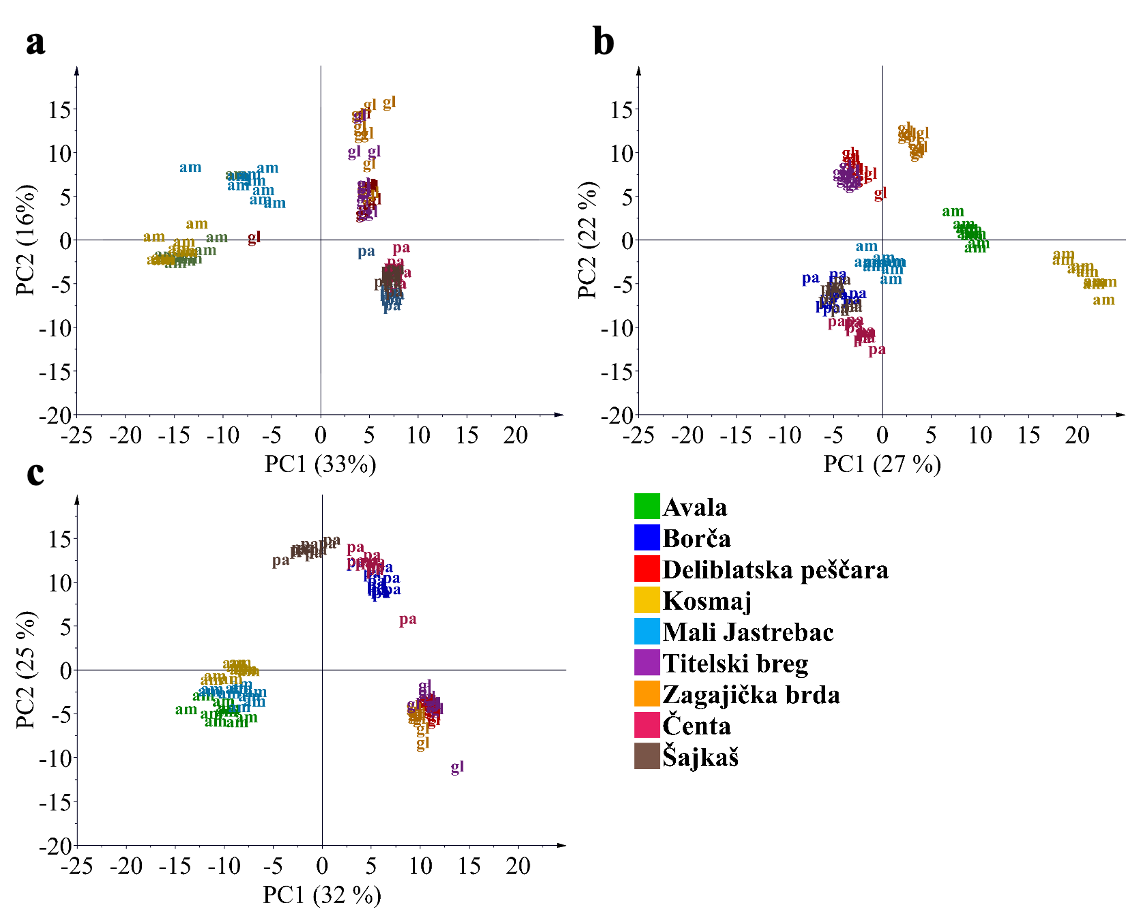


**Supplementary Fig. 1.** Principal component analysis of ^1^H NMR spectra latex, leaves, and roots of three *Euphorbia* species collected at different locations of Serbia. a, latex, b, leaves, c, roots. am, *Euphorbia amygdaloides*, gl, *Euphorbia glareosa*, pa, *Euphorbia palustris*. The separation of the samples by geographical origin is much more evident in leaves and much less evident in latexes.


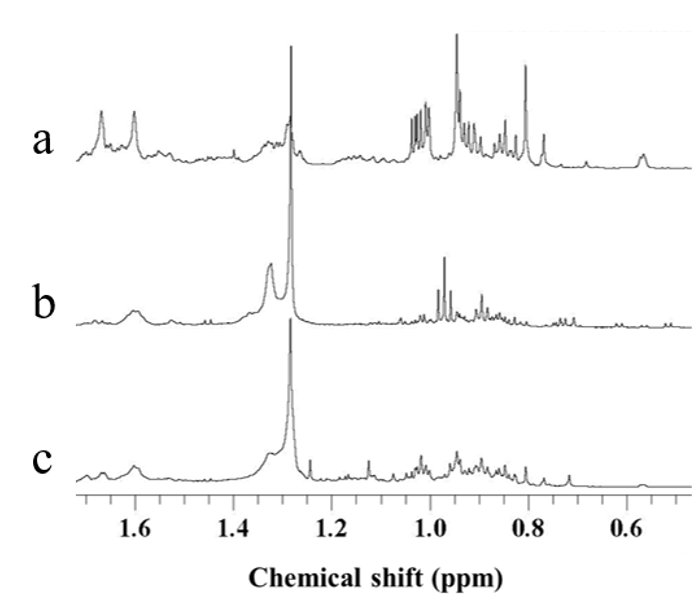


**Supplementary Fig. 2.** Representative ^1^H NMR spectra (600 MHz, MeOD + HMDSO) from latex, leaves, and roots in *Euphorbia amygdaloides*. a, latex, b, leaves, c, roots. Range of δ 0.4 – δ 1.8 of latex reveals a higher concentration of terpene resonances.


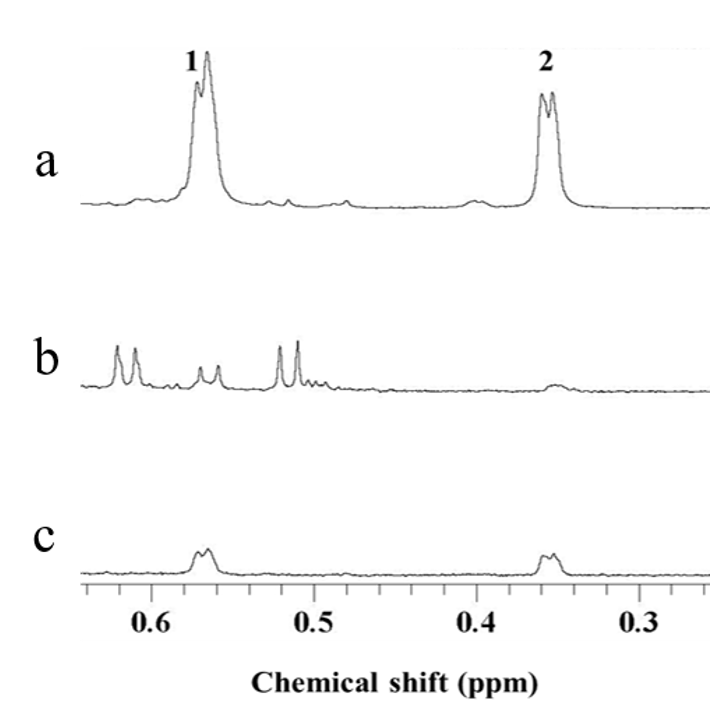


**Supplementary Fig. 3.** High field representative ^1^H NMR spectra (600 MHz, MeOD + HMDSO) from latex, leaves, and roots in *Euphorbia amygdaloides*. a, latex, b, leaves, c, roots. Two doublets at δ 0.55 (1) and δ 0.35 (2) correspond to the 19-*endo* and 19-*exo* protons, respectively, of the cyclopropane ring of 24-methylenecycloartanol with a concentration approximately eight times higher in latexes than in the other two tissues.


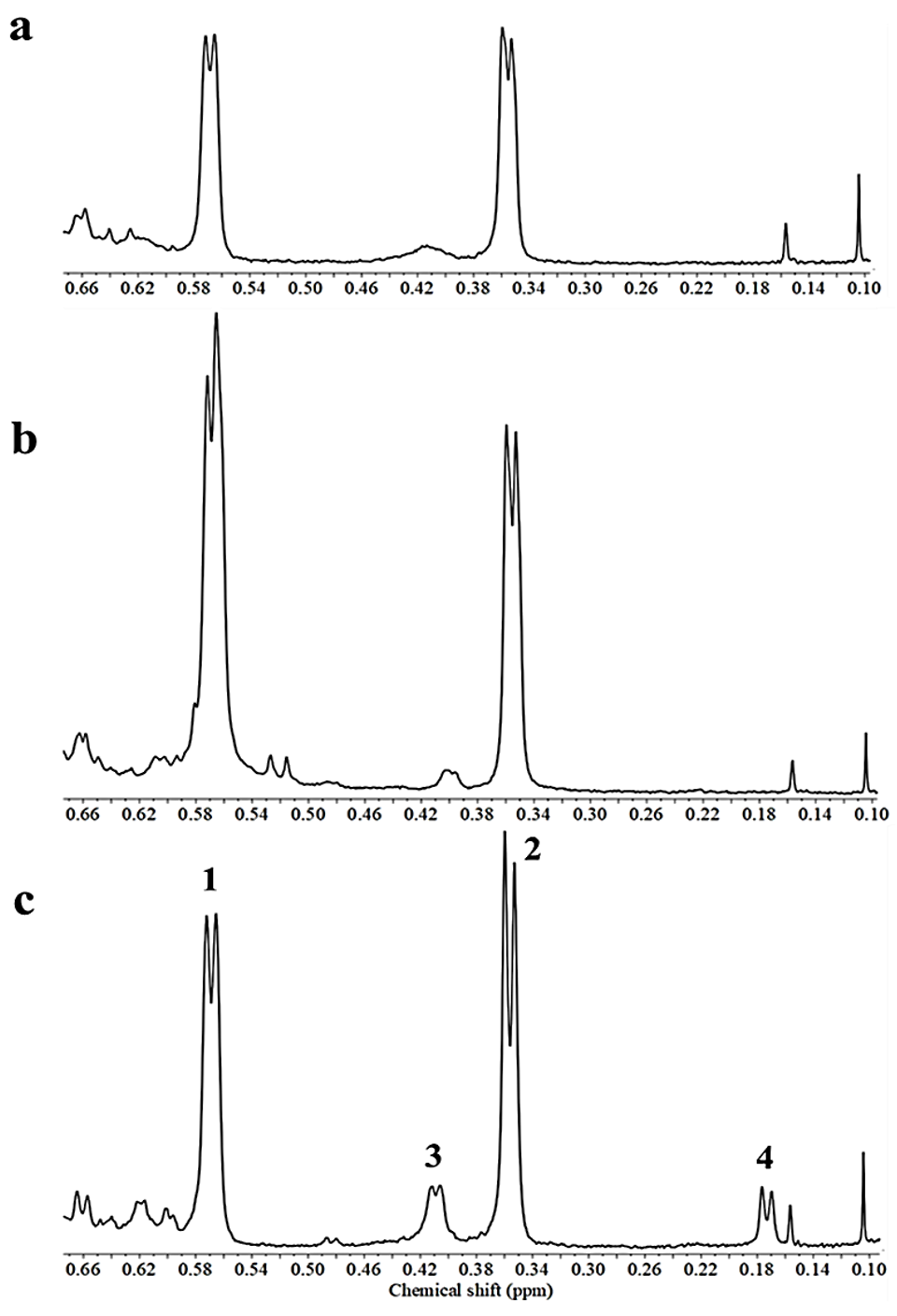


**Supplementary Fig. 4.** Content of 24-methylenecycloartanol and cycloeucalenyl acetate in the latex of three euphorbia species. a, *Euphorbia glareosa*, b, *Euphorbia amygdaloides*, c, *Euphorbia palustris*. 1, H-19*endo* from 24-methylenecycloartanol, 2, H-19*exo* from 24-methylenecycloartanol, 3, H-19*endo* from cycloeucalenyl acetate, 4, H-19*exo* from cycloeucalenyl acetate. Cycloeucalenyl acetate is distinctive metabolite of the latex of *E. palustris*.


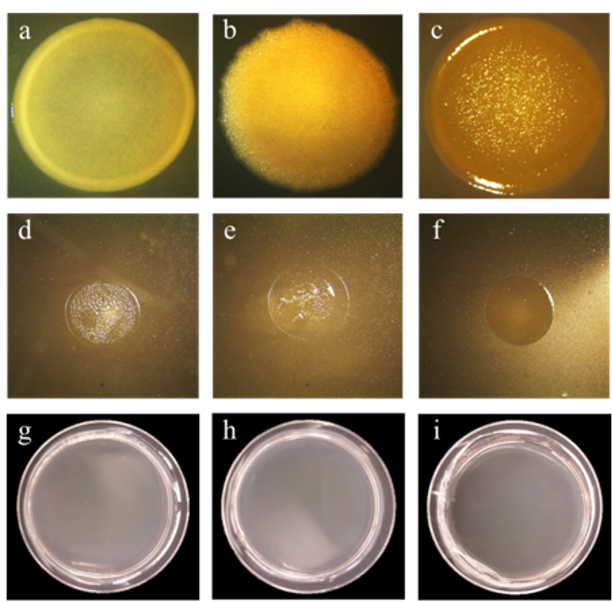


**Supplementary Fig. 5. Antibacterial effect of rubber against bacterial pathogens.** **a,** *Pseudomonas fluorescens,* **b,** *Pseudomonas putida*, **c,** *Pseudomonas viridiflava*. The first three images represent the negative control consisting of the bacteria growth in Mueller-Hinton agar (nutrient agar 2 for *P. viridiflava*) after 24 h; **d**, **e**, and **f**: the same bacteria growing on top of the rubber layer. The bacteria were not able to penetrate the layer and grow in the agar media; **g**, **h**, and **i**: growth control for the rubber treatment. The rubber layer with bacteria above it was removed, and the plates were incubated for 24 h to reveal possible bacterial growth.


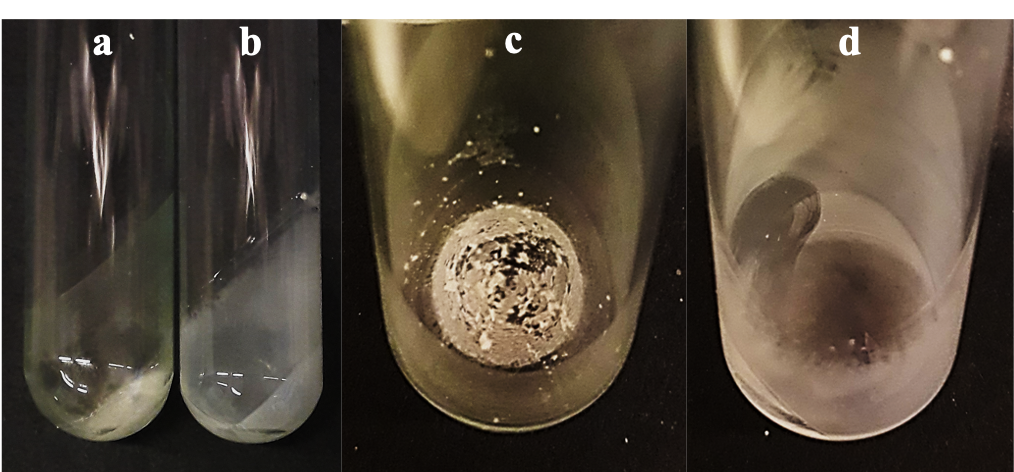


**Supplementary Fig. 6. Dispersive effect of polyisoprene.** Side view of **a,** test tube after vacuum centrifugation, which contained a mixture of 24-methylenecycloartanol and chloroform, **b,** a test tube after vacuum centrifugation, which contained a mixture of 24-methylenecycloartanol, chloroform and rubber. **c,** Front view of a test tube after vacuum centrifugation, which contained a mixture of 24-methylenecycloartanol and chloroform; the metabolite is deposited and crystalized at the bottom of the test tube. **d,** Front view of a test tube after vacuum centrifugation, which contained a mixture of 24-methylenecycloartanol, chloroform and rubber; the metabolite is dispersed through the rubber film around the tube wall.
